# Effect of dexamethasone in patients with ARDS and COVID-19 (REMED trial)—study protocol for a prospective, multi-centre, open-label, parallel-group, randomized controlled trial

**DOI:** 10.1186/s13063-021-05963-6

**Published:** 2022-01-15

**Authors:** Jan Maláska, Jan Stašek, František Duška, Martin Balík, Jan Máca, Jan Hruda, Tomáš Vymazal, Olga Klementová, Jan Zatloukal, Tomáš Gabrhelík, Pavel Novotný, Regina Demlová, Jana Kubátová, Jana Vinklerová, Adam Svobodník, Milan Kratochvíl, Jozef Klučka, Roman Gál, Mervyn Singer, on behalf of the REMED Study Group

**Affiliations:** 1 Department of Anaesthesiology and Intensive Care Medicine, Faculty of Medicine, University Hospital Brno and Masaryk University, Jihlavská 20, 625 00 Brno, Czech Republic; 2 Department of Anaesthesia and Intensive Care, 3rd Faculty of Medicine, University Hospital Královské Vinohrady and Charles University, Šrobárova, 1150 100 34 Prague, Czech Republic; 3 Department of Anaesthesia and Intensive Care, 1st Faculty of Medicine, General University Hospital in Prague and Charles University, U Nemocnice 499/2, 128 08 Prague, Czech Republic; 4 Department of Anaesthesiology and Intensive Care Medicine, Faculty of Medicine, University Hospital Ostrava and University Ostrava, 17. listopadu 1790, 708 52 Ostrava-Poruba, Czech Republic; 5 Department of Anaesthesiology and Intensive Care Medicine, Faculty of Medicine, St. Anne’s University Hospital and Masaryk University, Pekařská 664/53, 656 91 Brno, Czech Republic; 6 Department of Anaesthesiology and Intensive Care Medicine, 2nd Faculty of Medicine, University Hospital Motol and Charles University, V Úvalu 84/1, 150 06 Prague, Czech Republic; 7 Department of Anaesthesiology and Intensive Care Medicine, Faculty of Medicine, University Hospital Olomouc and Palacky University, I. P. Pavlova 185/6, 779 00 Olomouc, Czech Republic; 8 Department of Anaesthesiology and Intensive Care Medicine, Faculty of Medicine in Pilsen, University Hospital Plzeň and Charles University, alej Svobody 80, 304 60 Plzeň-Lochotín, Czech Republic; 9 Department of Anaesthesiology and Intensive Care Medicine, Tomáš Baťa Regional Hospital, Havlíčkovo nábřeží 600, 762 75 Zlín, Czech Republic; 10 Department of Anaesthesiology and Intensive Care, 1st Faculty of Medicine, Military University Hospital Praha and Charles University, U Vojenské nemocnice 1200, 169 02 Prague, Czech Republic; 11 Department of Pharmacology/CZECRIN, Faculty of Medicine, Masaryk University, Kamenice 5, 62500 Brno, Czech Republic; 12 Department of Paediatric Anaesthesiology and Intensive Care Medicine, Faculty of Medicine, University Hospital Brno and Masaryk University, Jihlavská 20, 625 00 Brno, Czech Republic; 13 Bloomsbury Institute of Intensive Care Medicine, Division of Medicine, University College London, Gower Street, London, WC1E 6BT UK

**Keywords:** COVID-19, Randomised controlled trial, Protocol, ARDS, Dexamethasone, Ventilator-free days

## Abstract

**Background:**

Since December 2019, SARS-CoV-2 virus has infected millions of people worldwide. In patients with COVID-19 pneumonia in need of oxygen therapy or mechanical ventilation, dexamethasone 6 mg per day is currently recommended. However, the dose of 6 mg of dexamethasone is currently being reappraised and may miss important therapeutic potential or may prevent potential deleterious effects of higher doses of corticosteroids.

**Methods:**

REMED is a prospective, open-label, randomised controlled trial testing the superiority of dexamethasone 20 mg (dexamethasone 20 mg on days 1–5, followed by dexamethasone 10 mg on days 6–10) vs 6 mg administered once daily intravenously for 10 days in adult patients with moderate or severe ARDS due to confirmed COVID-19. Three hundred participants will be enrolled and followed up for 360 days after randomization. Patients will be randomised in a 1:1 ratio into one of the two treatment arms. The following stratification factors will be applied: age, Charlson Comorbidity Index, CRP levels and trial centre. The primary endpoint is the number of ventilator-free days (VFDs) at 28 days after randomisation. The secondary endpoints are mortality from any cause at 60 days after randomisation; dynamics of the inflammatory marker, change in WHO Clinical Progression Scale at day 14; and adverse events related to corticosteroids and independence at 90 days after randomisation assessed by the Barthel Index. The long-term outcomes of this study are to assess long-term consequences on mortality and quality of life at 180 and 360 days. The study will be conducted in the intensive care units (ICUs) of ten university hospitals in the Czech Republic.

**Discussion:**

We aim to compare two different doses of dexamethasone in patients with moderate to severe ARDS undergoing mechanical ventilation regarding efficacy and safety.

**Trial registration:**

EudraCT No. 2020-005887-70. ClinicalTrials.gov NCT04663555. Registered on December 11, 2020

## Administrative information

Note: the numbers in curly brackets in this protocol refer to SPIRIT checklist item numbers. The order of the items has been modified to group similar items (see http://www.equator-network.org/reporting-guidelines/spirit-2013-statement-defining-standard-protocol-items-for-clinical-trials/).

**Table:** 

Title {1}	Effect of dexamethasone in patients with ARDS and COVID-19 (REMED trial)—study protocol for a prospective, multi-centre, open-label, parallel-group, randomized controlled trial
Trial registration {2a and 2b}.	EudraCT No.:2020-005887-70 and ClinicalTrials.gov Identifier: NCT04663555 on December 11, 2020, on ClinicalTrials.gov
Protocol version {3}	1.2 | 22.03.2021
Funding {4}	REMED is an investigator-initiated clinical trial. Funding will be granted from the project research infrastructure Czech Clinical Research Infrastructure Network CZECRIN (LM 2018128) and University Hospital Brno.
Author details {5a}	Jan Maláska^1*^, Jan Stašek^1^, František Duška^2^, Martin Balík^3^, Jan Máca^4^, Jan Hruda^5^, Tomáš Vymazal^6^, Olga Klementová^7^, Jan Zatloukal^8^, Tomáš Gabrhelík^9^, Pavel Novotný^10^, Regina Demlová^11^, Jana Kubátová^11^, Jana Vinklerová^11^, Adam Svobodník^11^, Milan Kratochvíl^12^, Jozef Klučka^12^, Roman Gál^1^, Mervyn Singer^13^ on behalf of the REMED Study Group. ^1^Department of Anaesthesiology and Intensive Care Medicine, University Hospital Brno and Masaryk University, Faculty of Medicine, Jihlavská 20, 625 00 Brno, Czech Republic, Jan Maláska: malaska.jan@fnbrno.cz, Jan Stašek: stasek.jan@fnbrno.cz, Roman Gál: gal.roman@fnbrno.cz ^2^Department of Anaesthesia and Intensive Care, University Hospital Královské Vinohrady and Charles University, 3rd Faculty of Medicine, Šrobárova 1150 100 34 Praha, Czech Republic, frantisek.duska@lf3.cuni.cz ^3^Department of Anaesthesia and Intensive Care, General University Hospital in Prague and Charles University, 1st Faculty of Medicine, U Nemocnice 499/2 128 08 Praha, Czech Republic, martin.balik@vfn.cz ^4^Department of Anaesthesiology and Intensive Care Medicine, University Hospital Ostrava and University Ostrava, Faculty of Medicine, 17. listopadu 1790, 708 52 Ostrava-Poruba, Czech Republic, jan.maca@fno.cz ^5^Department of Anaesthesiology and Intensive Care Medicine, St. Anne’s University Hospital and Masaryk University, Faculty of Medicine, Pekařská 664/53, 656 91 Brno, Czech Republic, jan.hruda@fnusa.cz ^6^Department of Anaesthesiology and Intensive Care Medicine, University Hospital Motol and Charles University, 2nd Faculty of Medicine, V Úvalu 84/1, 150 06 Praha 5, Czech Republic, tomas.vymazal@fnmotol.cz ^7^Department of Anaesthesiology and Intensive Care Medicine, University Hospital Olomouc and Palacky University, Faculty of Medicine, I. P. Pavlova 185/6, 779 00 Olomouc, Czech Republic, Olga.Klementova@fnol.cz ^8^Department of Anaesthesiology and Intensive Care Medicine, University Hospital Plzeň and Charles University, Faculty of Medicine in Pilsen, alej Svobody 80, 304 60 Plzeň-Lochotín, Czech Republic, ZATLOUKALJ@fnplzen.cz ^9^Department of Anaesthesiology and Intensive Care Medicine, Tomáš Baťa Regional Hospital, Havlíčkovo nábřeží 600, 762 75 Zlín, Czech Republic, Tomas.Gabrhelik@bnzlin.cz ^10^Department of Anaesthesiology and Intensive Care, Military University Hospital Praha and Charles University, 1st Faculty of Medicine, U Vojenské nemocnice 1200, 169 02 Praha, Czech Republic, Pavel.Novotny@uvn.cz ^11^Department of Pharmacology/CZECRIN, Masaryk University, Faculty of Medicine, Kamenice 5, Brno, 62500 Czech Republic, Regina Demlová: demlova@med.muni.cz, Jana Kubátová: jana.kubatova@med.muni.cz, Jana Vinklerová: jvinkler@med.muni.cz, Adam Svobodník: svobodnika@gmail.com ^12^Department of Paediatric Anaesthesiology and Intensive Care Medicine, University Hospital Brno and Masaryk University, Faculty of Medicine, Jihlavská 20, 625 00 Brno, Czech Republic, Milan Kratochvíl: kratochvil.milan@fnbrno.cz, Jozef Klučka: klucka.jozef@fnbrno.cz ^13^Bloomsbury Institute of Intensive Care Medicine, Division of Medicine, University College London, Gower Street, London, WC1E 6BT, United Kingdom, m.singer@ucl.ac.uk *corresponding author
Name and contact information for the trial sponsor {5b}	University Hospital Brno Jihlavská 20 625 00 Brno, Czech Republic
Role of sponsor {5c}	Trial funders have no role in the study design, collection, analysis and interpretation of the data. Investigators declare no financial or non-financial competing interest regarding the focus of this trial.

## Clinical trial background

### Introduction

Since December 2019, SARS-CoV-2 virus has infected millions of people worldwide. A significant number of patients develop a hyperinflammatory state affecting the lungs, which may lead to the need for oxygen therapy. In most severe cases, acute respiratory distress syndrome (ARDS) develops, and high flow oxygen therapy or invasive mechanical ventilation is necessary [1]. Therapeutic options in coronavirus disease 2019 (COVID-19)-associated ARDS patients remain limited, and mortality is still excessive.

### Rationale and up-to-date evidence

Systemic corticosteroids have the potential to limit hyperinflammatory response by modulating the immune system. This effect is mediated mainly by binding to the glucocorticoid receptor (GR) α [2]. Their effectiveness was proved in heterogeneous ARDS patients recently [3].

In patients with COVID-19 pneumonia in need of oxygen therapy or mechanical ventilation, dexamethasone 6 mg per day is currently recommended. This therapy is mandated by the results of the RECOVERY trial [4]. After this trial was published, three randomized trials comparing hydrocortisone [5, 6] or dexamethasone [7] against placebo were stopped prematurely. All these studies were included in the subsequent individual patients’ data (IPD) meta-analysis [8]. However, the dose of 6 mg of dexamethasone is currently being reappraised.

The aforementioned study in non-COVID-19 ARDS patients [3] used 20 mg of dexamethasone per day, which is roughly equivalent to the methylprednisolone regimen (1 mg/kg/day) studied in early severe ARDS patients [9]. Only these moderate doses (80–100 mg of methylprednisolone, equivalent to 15–19 mg of dexamethasone) have the full potential to modulate the immune response by saturating GRα receptors [2]. Importantly, prematurely stopped CoDEX trial [7] comparing dexamethasone against placebo in COVID-19 ARDS patients used an initial daily dose of 20 mg of dexamethasone vs placebo.

In light of these facts, 6 mg of dexamethasone given to COVID-19 patients with different severities of illness (WHO classification groups 5–10) may miss important therapeutic potential or may prevent potential deleterious effects of a full-dose therapeutic corticosteroid. The authors hypothesize that the patients with moderate to severe ARDS undergoing mechanical ventilation may benefit from higher doses of dexamethasone [3, 7, 9].

### Anticipated risks and benefits

There may or may be no benefit from a higher dose of dexamethasone in the improvement of the clinical outcomes of an individual participant. However, there is a potential benefit to the society from the participation in this study resulting from insights gained about the dexamethasone use and posology. The potential risks of participating in this study are those associated with dexamethasone use, i.e. known adverse effects of potent glucocorticoids.

## Trial objectives and endpoints

### Primary objective(s) and endpoint(s)

The primary objective of this study is to test the hypothesis that administration of dexamethasone 20 mg is superior over 6 mg in adult patients with moderate or severe ARDS due to confirmed COVID-19.

The primary endpoint is the number of ventilator-free days (VFDs) at 28 days after randomization.

VFDs are defined as being alive and free from mechanical ventilation (more than 48 h):

- Day 0 is the day of randomization.
- The time frame is 28 days.
- Free from mechanical ventilation means extubation for > 48 h without reintubation in a 28-day survivor or disconnection from the ventilator in patients with tracheostomy (irrespective of PEEP valve) for > 48 h without reconnection to the ventilator in a 28-day survivor.
- VFDs are counted from the last successful extubation or disconnection from the ventilator.
- If death occurs before day 28, VFD = 0 (to penalize non-survival, regardless of intubation status).
- If death occurs after day 28, 28-day ventilation and survival status is used for calculating VFDs (censor after 28 days).

### Secondary objective(s) and endpoint(s)

The secondary objective is to investigate the efficacy and safety of dexamethasone 20 mg vs dexamethasone 6 mg.

The following are the secondary endpoints:

- Mortality from any cause at 60 days after randomization
- Dynamics of inflammatory marker (CRP) change from day 1 to day 14
- WHO Clinical Progression Scale at day 14 (range 0–10; 0 = no illness, 1–9 = increasing level of care and 10 = death) [11]
- Adverse events related to corticosteroids (new infections, new thrombotic complications) until day 28 or hospital discharge
- Independence at 90 days after randomization assessed by the Barthel Index

### Exploratory objective(s) and endpoint(s)

The exploratory objective of this study is to assess the long-term consequences on mortality and quality of life at 180 and 360 days.

## Trial design

REMED is a prospective phase II open-label randomized controlled trial testing the superiority of dexamethasone 20 mg vs 6 mg. The study is multi-centre and will be conducted in intensive care units (ICUs) of university hospitals in the Czech Republic. The trial aims to be pragmatic, i.e. designed to evaluate the effectiveness of the intervention in conditions that are very close to the real-life routine clinical practice. Dexamethasone will be administered once daily intravenously for 10 days. Three hundred participants will be enrolled and followed up for 360 days after randomization.

## Trial population

### Inclusion criteria

Subjects will be eligible for the trial if they meet all of the following criteria:

1. Adult (≥ 18 years of age) at time of enrolment
2. Present COVID-19 (infection confirmed by RT-PCR or antigen testing)
3. Intubation/mechanical ventilation or ongoing high-flow nasal cannula (HFNC) oxygen therapy
4. Moderate or severe ARDS according to the Berlin criteria:

- Moderate—PaO_2_/FiO_2_ 100–200 mmHg
- Severe—PaO_2_/FiO_2_ < 100 mmHg

5. Admission to ICU in the last 24 h

### Exclusion criteria

Subjects will not be eligible for the trial if they meet any of the following criteria:

1. Known allergy/hypersensitivity to dexamethasone or excipients of the investigational medicinal product (e.g. parabens, benzyl alcohol)
2. Fulfilled criteria for ARDS for ≥ 14 days at enrolment
3. Pregnancy or breastfeeding
4. Unwillingness to comply with contraception measurements from the enrolment to at least 1 week after the last dose of dexamethasone (sexual abstinence is considered as the adequate contraception method)
5. End-of-life decision or patient is expected to die within next 24 h
6. Decision not to intubate or ceilings of treatment in place
7. Immunosuppression and/or immunosuppressive drugs in medical history:

- Systemic immunosuppressive drugs or chemotherapy in the past 30 days
- Systemic corticosteroids use before hospitalization
- Corticosteroid administration (dexamethasone equal or less than 8 mg per day or other corticosteroids in equivalent dose) during the present hospital stay for COVID-19 for more than last 5 days before enrolment
- Systemic corticosteroids during the present hospital stay for other conditions than COVID-19 (e.g. septic shock)
- Dexamethasone more than 8 mg per day or other corticosteroids in equivalent dose during the present hospital stay for COVID-19 for more than one single dose

8. Present haematological or generalized solid malignancy
9. Any of the following contraindications of corticosteroids:

- Intractable hyperglycaemia
- Active gastrointestinal bleeding
- Adrenal gland disorders
- The presence of superinfection diagnosed with locally established clinical and laboratory criteria without adequate antimicrobial treatment

10. Cardiac arrest before ICU admission
11. Participation in another interventional trial in the last 30 days

### Randomization and stratification

Randomization will be carried out within the electronic case report form (eCRF) by the stratified permuted block randomization method. The allocation sequences will be prepared by a statistician independent of the study team. Allocation to the treatment arm of an individual patient will not be available to the investigators before the completion of the whole randomization process. Following stratification factors will be applied:

- Age < 65 and ≥ 65 [12]
- Charlson Comorbidity Index (CCI) < 3 and ≥ 3
- CRP < 150 mg/L and ≥ 150 mg/L
- Trial centre

Patients will be randomized in a 1:1 ratio in one of the two treatment arms. Randomization through eCRF will be available 24 h every day.

### Blinding

This is an open-label trial, in which the participants and the study staff will learn about the allocated intervention. Blinded pre-planned statistical analysis will be performed [13] according to the “Statistics” section.

### Enrolment stopping rules

Enrolment of new subjects into the trial will be stopped if any of the following is encountered until the sponsor determines if it is safe for the trial to continue enrolment:

1. Death that is related to dexamethasone.
2. SAE or SUSAR that is related to dexamethasone during 28 days post-administration.
3. Emergence of new data that may lead to the trial becoming unethical. This may be merely due to safety concerns of the interventional group (e.g. if harm is reported by other trials).
4. After the interim analysis, the steering committee will review the primary outcomes and the summary of adverse events in both arms whilst still blinded to the treatment allocation. The enrolment can be stopped if the following criteria are fulfilled:
  - There is a significant difference in the primary outcome at *p* < 0.01 between the arms.
  - Futility: the futility criterion is not binding for the steering committee. Based on available data, the study statistician calculates the probability of being able to reject the null hypothesis by achieving the target number of subjects and the probability of type II error made by stopping the trial prematurely.

### Premature termination of participation in the trial

Reasons for the early termination of a patient’s participation include the following:

- Withdrawal of informed consent (subject’s decision to withdraw for any reason)
- Important deviation in the process of informed consent
- Life-threatening adverse reaction to dexamethasone at the discretion of the investigator
- Newly emerged pregnancy of a participant after the enrolment

Subjects can terminate their participation prematurely at any time at their request for any reason, but they must notify the investigator. The investigator must contact the sponsor to report the premature discontinuation.

The investigator must:

- Instruct the participant about the right on early termination of participation
- Assure him/her that the end of participation will not affect the attitude of the physician or further treatment and its quality
- Ask for discussing this decision with the investigator in advance
- Make a note in the patient’s medical records and eCRF about the date of early termination of participation

The sponsor reserves the right to discontinue the study at any time if there is a significant safety concern (e.g. grade 4 adverse reactions to dexamethasone) or insufficient recruitment despite intensified efforts to enrol patients or if repeated poor study documentation occurs at a site. The trial can be discontinued by the decision of the regulatory authority or ethics committee, as well.

## Study treatment

### Dexamethasone (ATC code: H02AB02)

Dexamethasone solution for injection/infusion is the investigational medicinal product as well as the comparator. The trial will assess two doses, 20 mg (investigational) vs 6 mg (comparator).

**Table:** 

All authorized medicinal products containing dexamethasone in the form of solution for i.v. injection/infusion can be used.	

#### Qualitative and quantitative composition

The active pharmaceutical ingredient is dexamethasone sodium phosphate (4 mg/mL).

The excipients differ in various dexamethasone solutions for inj./inf. Please see the SmPC of the medicinal product you are using. Several excipients have known biological effects (e.g. allergic reactions): methylparaben, propylparaben and benzyl alcohol. Allergy or hypersensitivity to dexamethasone or excipients is an exclusion criterion.

#### Pharmaceutical form and route of administration

Solution for injection/infusion; intravenous administration

#### Other information

Detailed pharmacological profile of the drug, including information on the mechanism of action, contraindications, drug interactions, adverse effects and others, can be found in the Summary of Product Characteristics (SmPC).

### Packaging and labelling

The medicinal product will be labelled in accordance with applicable legislation.

### Delivery and storage

The medicinal product containing dexamethasone will be ordered regularly by the trial sites’ hospital pharmacies and taken from the Czech market. It will be stored up to 30 °C in the secondary package to keep it from the light according to the SmPC recommendations.

### Dosing schedule

Patients in the intervention group will receive dexamethasone 20 mg intravenously once daily on days 1–5, followed by dexamethasone 10 mg intravenously once daily on days 6–10. Patients in the control group will receive dexamethasone 6 mg days 1–10.

### Dose modification and delays

For this study, no dose modification or delays are possible, except for stopping dexamethasone administration if the patient is being discharged from the hospital due to clinical improvement before day 10.

Previous treatment with dexamethasone more than 8 mg per day or other corticosteroids in an equivalent dose during the present hospital stay for COVID-19 for more than one single dose is an exclusion criterion. If the study subject is treated with dexamethasone 8 mg or less per day or other corticosteroids in an equivalent dose less than 5 days before the enrolment, he/she can be enrolled, and there will be no changes in the duration of study drug administration.

### Permitted concomitant medication

Study participants will receive the best standard of care according to the local protocols and national guidelines, including the state-of-the-art anti-infective and adjunctive treatment of COVID-19, sedation and delirium management, physiotherapy, ventilator and haemodynamic management.

Currently, the standard of care for hospitalized patients with COVID-19 that require oxygen support [14] can consist of the following:

- Remdesivir (at the discretion of the investigator)
- Prevention of thromboembolic disease (anticoagulants)
- Gastric ulcer prophylaxis for patients on dexamethasone
- Nutrition, hydration, electrolytes, vitamins and glucose administration
- Antipyretics (paracetamol, metamizole)
- Antibiotics
- Bronchodilators

All chronic medication prescribed to the patient before the enrolment is permitted to continue with the exceptions defined in the “Exclusion criteria” and “Restricted concomitant medication” sections. All necessary medication needed during the trial is allowed with exceptions defined in the “Restricted concomitant medication” section.

### Restricted concomitant medication

Systemic corticosteroids other than dexamethasone are restricted, as well as other investigational medicinal products currently tested for the treatment of COVID-19 (e.g. investigational antivirals or monoclonal antibodies). Remdesivir is not considered as an investigational drug, as it has already been authorized.

Concomitant treatment with potent inhibitors of CYP3A increases the risk of systemic adverse effects of dexamethasone. In cases where the benefits of such treatment do not overweight this increased risk, combination with strong inhibitors of CYP3A is considered restricted.

**Table:** 

Potent inhibitors of CYP3A include, e.g., systemic azole antimycotics, clarithromycin, erythromycin, telithromycin, diltiazem and verapamil.	

Concomitant use of potent inducers of CYP3A decreases the effect of dexamethasone, which may then bias the results. In cases where benefits of such treatment do not overweight this risk of bias, combination with potent inducers of CYP3A is considered restricted.

**Table:** 

Potent inducers of CYP3A include, e.g., phenytoin, rifampicin, phenobarbital and carbamazepine.	

Detailed information on drug interactions could be found in SmPC.

## Course of the trial

### Screening of eligible patients

Patients admitted to the ICU of each centre will be screened daily for eligibility, and all the patients will be recorded in the screening log of eCRF. For patients with the completion of consent, appropriate enrolment information will be recorded to eCRF and only after all required information are provided the randomization and allocation will proceed.

For details on obtaining the informed consent, see The “Informed consent procedure” section.

### Overview of medical care about the study participant

Patients in both arms will receive the best standard of care. General supportive care guided by the official recommendations and local protocols will be given, as described in the “Permitted concomitant medication” section.

The centres are encouraged to follow appropriate antibiotic stewardship practices, e.g. guidelines for hospital-acquired and ventilator-associated pneumonia (VAP/HAP) management [15]. As for ventilation strategies, the centres are encouraged to comply with the best evidence of ventilator settings for ARDS patients, i.e. tidal volume approx. 6 mL/kg of predicted body weight, driving pressure (∆P) < 15 cm of water, plateau pressure (Ppl) < 30 cm of water, setting inspiratory fraction of oxygen (FiO_2_) and positive end-expiratory pressure (PEEP) to keep SaO_2_ ≥ 90% or PaO_2_ ≥ 8 kPa and set respiratory rate (RR) to maintain pH ≥ 7.20. Besides ventilator settings, other measures are left to the local protocols and at the discretion of each centre, e.g. prone positioning, neuromuscular blocking agents, lung recruitment manoeuvres and extracorporeal membrane oxygenation (ECMO).

Weaning from mechanical ventilation is performed by the daily screening of readiness through using the spontaneous breathing trial (SBT). If SBT is successful and no other significant reason against extubation is present, the patient is extubated. Patients allowed spontaneous ventilation while on ECMO are classified as support dependent until ECMO is weaned off.

### Clinical examinations and assessments

During the trial course, subjects will be monitored during hospitalization and after discharge. A summary of scheduled procedures is outlined in the “Study procedures” section. For a graphic summary, see Table 1.

**Table 1:**
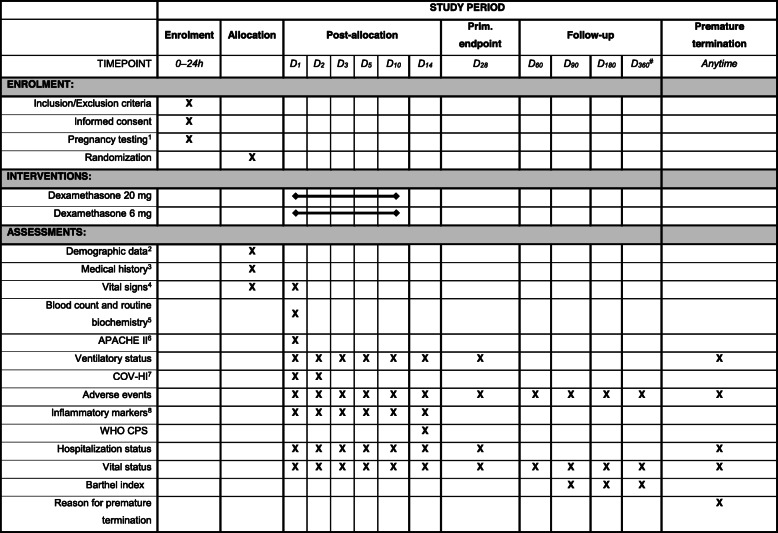
Clinical trial schedule

^1^hCG from a peripheral blood sample in women of childbearing potential; if statim hCG is not available, perform urine pregnancy testing

^2^Age, sex, race or ethnic group and BMI

^3^Allergies, comorbidities, and Charlson Comorbidity Index (CCI) and chronic medication

^4^Temperature, blood pressure, heart rate and respiratory rate

^5^Blood count (RBC, WBC, haematocrit, lymphocyte count, platelets), coagulation profile, renal and liver functions (BUN, creatinine, bilirubin, AST, ALT), d-dimer, LDH, troponin, pH, blood gases, sodium, potassium, chloride and glycaemia (other laboratory tests and imaging methods are left at the discretion of each centre)

^6^Counted using the worst values in the first 24 h of ICU stay

^7^*D*_*1*_, CRP, ferritin; *D*_*2*_, CRP; COV-HI is defined as CRP ≥ 150 mg/L or doubling within 24 h from greater than 50 mg/L, or ferritin ≥ 1500 μg/L [11, 15, 16]

^8^CRP (other markers, e.g. procalcitonine, presepsin, interleukin-6 at the discretion of each centre)

^#^End of trial visit (or phone call)

### Study procedures

At enrolment:

- Eligibility screen—inclusion and exclusion criteria
- Informed consent procedure
- Pregnancy testing (hCG) in women with childbearing potential—preferentially from a blood sample, if statim hCG is not available at the time of enrolment and urine pregnancy testing is necessary
- Demographic data (age, sex, race or ethnic group, BMI)
- Medical history (allergies, comorbidities and CCI, chronic medication)
- Vital signs (temperature, blood pressure, heart rate, respiratory rate)
  Day 1:
- Vital signs (temperature, blood pressure, heart rate, respiratory rate)
- Administration of dexamethasone according to the allocation to treatment arms
- Blood count and routine biochemistry [RBC, WBC, haematocrit, lymphocyte count, platelets, coagulation profile, renal and liver functions (BUN, creatinine, AST, ALT, bilirubin), d-dimer, LDH, troponin, pH, blood gases, sodium, potassium, chloride, glycaemia]
- Inflammatory markers (CRP; other markers, e.g. procalcitonine, presepsin, interleukin-6 at the discretion of each centre)
- COV-HI assessment—based on day 1 CRP or ferritin level
- APACHE II score counted using the worst values in the first 24 h of ICU stay (the score is determined based on specific parameters from medical history, vital signs, blood count and biochemistry, and other examinations)
  Day 2:
- Administration of dexamethasone according to the allocation to treatment arms
- Inflammatory markers (CRP; other markers, e.g. procalcitonine, presepsin, interleukin-6 at the discretion of each centre)
- COV-HI assessment—based on day 2 CRP level
  Day 3 to day 10:
- Administration of dexamethasone according to the allocation to treatment arms
- Inflammatory markers (CRP; other markers, e.g. procalcitonine, presepsin, interleukin-6 at the discretion of each centre)
  Day 14:
- World Health Organization Clinical Progression Scale
- Inflammatory markers (CRP; other markers, e.g. procalcitonine, presepsin, interleukin-6 at the discretion of each centre)
  Day 1 to day 28/ICU discharge (whichever comes first):
- Checking adverse events and adverse reactions (with a special interest in newly emerged infections and new thrombotic complications)
- Ventilatory status, i.e. use of a mechanical ventilator or other ventilation/oxygen support
- Vital status
- Discharge from ICU and hospital
  Days 60, 90, 180 and 360:
- Out-patient visits (if possible) or structured phone call interviews led by study nurse with the patient or his/her family member or caregiver
- Vital status
- Quality of life by the assessment of daily life activities and functional independence (Barthel Index)
- Checking adverse events and reactions

### Biological samples

Blood samples will be taken following local practice and analysed in the respective clinical laboratory facility of the trial centre. No other biological specimens will be obtained or stored in this trial.

### Assessment tools, scores and scales

The following tools will be used during the trial:

- APACHE II
- Barthel Index
- Charlson Comorbidity Index
- COV-HI
- Glasgow Coma Scale
- Spontaneous breathing trial
- WHO CPS

## Safety assessments

Before the start of the clinical trial, all investigators will undergo training in pharmacovigilance requirements and obtain detailed written instructions for recording and reporting of AEs. These instructions for pharmacovigilance will be part of the investigator’s site file.

### Definitions (according to the Directive 2001/20/EG)

For clinical trials, effective legislation has introduced the following definitions, see Table 2.

**Table 2:** Definitions of safety signals in clinical trials

Adverse event	AE	Any untoward medical occurrence in a patient or clinical trial subject administered a medicinal product and which does not necessarily have a causal relationship with this treatment
**Adverse drug reaction**	ADR	All untoward and unintended responses to an investigational medicinal product related to any dose administered
**Serious adverse event**	SAE	A serious adverse event/reaction is any untoward medical occurrence or effect that at any dose: • **Results in death** • **Is life-threatening** • **Requires hospitalization or extension of existing hospitalization** • **Results in persistent or significant disability or incapacity** • **Is a congenital anomaly or birth defect**
**Serious adverse reaction**	SADR	
**Unexpected adverse reaction**	UADR	Adverse reaction, the nature, severity or outcome of which is not consistent with the product information (SmPC)
**Suspected unexpected serious adverse reaction**	SUSAR	Any suspected adverse reaction related to the study treatment that is both serious and unexpected

### AE surveillance, recording and documentation

AE could be diseases or symptoms which occur or worsen after the enrolment of a patient in the clinical trial. All AEs need to be documented, no matter if the investigator suspects a causal connection to the study medication. AE will be monitored and documented from the day of giving informed consent until the end of participation in the study (i.e. day 360).

Subjects will be instructed to report any AEs that they experience to the investigator. The investigator should actively ask about AEs.

Each AE should be described, documented in the eCRF and evaluated to determine the following:

- Seriousness
- Severity
- Causality, i.e. relation to the study medication
- Duration (start and end dates or whether it continues)
- Action taken (no action taken, study medication discontinued, prolongation of the ongoing hospitalization, administration of a drug, etc.)
  AE needs to be followed until its resolution, i.e. until it subsides, stabilizes and becomes chronic or the subject dies.
  If AE fulfils the criteria of SAE, a separate form must be completed besides the standard eCRF record.
  AEs will be recorded according to the Medical Dictionary for Regulatory Activities (MedDRA). The most recent MedDRA version at the start of the study will be used.

### Treatment of AE

A patient with an AE must receive appropriate therapy. The investigator may decide to discontinue the study medication. The patient will remain under medical supervision until the investigator believes that the AE has been resolved.

### Assessment of seriousness

AE is considered serious if it fulfils the definition in the “Definitions (according to the Directive 2001/20/EG)” section.

Some situations can be considered as SAE even if they do not fulfil the criteria of the definition. These are important medical events that may not be immediately life-threatening, or result in death, or hospitalization but may jeopardize the subject, or may require intervention to prevent one of the other outcomes listed in the definition above. These should also be considered SAE—e.g. allergic bronchospasm, convulsions or other states requiring treatment.

If AE fulfils the criteria of SAE, a separate serious adverse event form must be completed and sent to the sponsor. For the procedure, see the “Reporting of SAE and SUSAR” section.

In this clinical trial, the following SAEs are excluded from the notification requirement:

- SAE which occurs after enrolment (i.e. after giving informed consent), but before the study medication was initiated
- Hospitalization(s) planned before the enrolment
- Death not related to the dexamethasone use^1^

### Assessment of intensity (severity)

The intensity (severity) of an AE should be evaluated according to these 5 categories:

**Table:** 

Grade 1	AE is asymptomatic or mildly symptomatic and requires only observation, no medical intervention.
**Grade 2**	AE with medium intensity, requires local, non-invasive or small-scale treatment.
**Grade 3**	AE is medically significant and requires hospitalization or extension of ongoing hospitalization, but it is not directly life-threatening.
**Grade 4**	AE is life-threatening and requires urgent significant medical intervention.
**Grade 5**	AE leads to death.

### Assessments of causality

To assess the relation between administration of the study medication and the AE, the following definitions apply:

- Related—The event is known to occur with the study medication, there is a reasonable possibility that the study medication caused the AE, or there is a temporal relationship between the study medication and AE. Reasonable possibility means that there is evidence to suggest a causal relationship between the study medication and the AE.
- Not related—There is no reasonable possibility that the administration of the study medication caused the AE, there is no temporal relationship between the study medication and AE onset, or an alternate aetiology has been established.

**Table:** 

The investigator is obliged to report any SAE within 24 h after he/she learns about it to the sponsor using serious adverse event form (SAE form). The blank forms are stored in the investigator’s site file. The announcement will be done by e-mail: farmakovigilance@med.muni.cz

### Reporting of SAE and SUSAR

If at that point all required information is not available, succeeding records will be sent by the investigator. The sponsor will check the notification completeness and formal plausibility. If required, queries will be made and followed up.

In the case of death, a copy of the autopsy record should be added. If the death of the subject complies with the definition of SUSAR (see the “Definitions (according to the Directive 2001/20/EG)” section), it will be reported as SUSAR (see below).

The sponsor has full responsibility for the safety of the clinical trial. Further reporting of AEs to the competent authorities according to the legal requirements is the responsibility of the sponsor.

#### Follow-up

All SAEs should be followed until their resolution, i.e. they subside, stabilize and become chronic, or the subject dies followed. In case of early termination because of SAE occurrence, the subject should be followed until SAE resolution. All necessary extra visits will be recorded in the eCRF as “unscheduled visits”.

Follow-up information is sent using a new SAE Form stating that this is a follow-up to the previously reported SAE and giving the date of the original report. Within 24 h of receipt of follow-up information, the investigator must update the SAE form and submit any supporting documentation (e.g. laboratory test reports, subject discharge summary, autopsy report) The follow-up information should describe whether the event has resolved or continues, if and how it was treated and whether the patient continued or withdrew from trial participation.

#### Reporting of SUSAR

SAE related to the study medication fulfilling the criterion of unexpectedness (i.e. SUSAR) must be reported by the sponsor to the Ethics Committee and the EudraVigilance database (module EVCTM), at the latest 15 days after it becomes known. The sponsor will also inform all investigators involved in the trial. Reporting to the EudraVigilance database will be performed via regulatory authority according to the agreement between the sponsor and the regulatory authority. In case of a fatal or life-threatening SUSAR, the sponsor will report all relevant information immediately, at the latest 7 days after the event becomes known. Any subsequent additional information is forwarded within the next 8 days if necessary.

### Pregnancy

Pregnant or breastfeeding women cannot be included in the study. Pregnancy testing (hCG, blood sample) is obligatory at enrolment in women of childbearing potential (i.e. from menarche to the onset of postmenopausal state). In case the information regarding menstrual cycle cannot be obtained from the patient due to her health or consciousness status, pregnancy testing is also necessary independently of the patient’s age. If statim hCG is not available at the moment of enrolment, investigators should perform urine pregnancy testing. Urine test strips will be supplied to the trial centres, where statim hCG is not available, e.g. on weekends or public holidays.

Participants, men and women, must comply with the required contraception measurements from the enrolment to at least 1 week after the last dose of dexamethasone. Sexual abstinence is considered as the adequate contraception method for this clinical trial. Newly emerged pregnancy in the hospitalization phase of the trial is highly unlikely. However, if it is diagnosed, dexamethasone treatment must be stopped. If a participant becomes pregnant during the follow-up phase, she must inform the investigator.

**Table:** 

The investigator should report the pregnancy to the sponsor within 24 h of learning of its occurrence using pregnancy form. This announcement will be done by e mail: farmakovigilance@med.muni.cz.	

Pregnancy should be followed by the investigator until completion. If it ends for any reason before the anticipated date, the investigator should notify the sponsor. At the completion of the pregnancy, the investigator will document the outcome of the pregnancy. If the outcome of the pregnancy meets the criteria for classification as SAE (e.g. spontaneous abortion, stillbirth, neonatal death, postpartum complication or congenital anomaly), the investigator should follow the procedures for reporting an SAE.

## Statistics

A separate statistical analysis plan (SAP) and statistical interim analysis plan (SIAP) will be prepared to provide details on the approach to analyses. The SIAP will be finalized before the interim database lock; the SAP will be finalized before the final database lock. All eventual deviations from the SAP will be described and justified in the relevant part of The Clinical Trial report.

### Sample size determination

The sample size was calculated to detect the difference of 3 VFDs at 28 days (primary efficacy endpoint) between the two treatment arms at two-sided type I error of 0.05 and power of 80%. Based on the data from a multi-centre randomized controlled trial in COVID-19 ARDS patients in Brazil [7] and the multi-centre observational study from French and Belgian ICUs regarding moderate to severe ARDS related to COVID-19 [18], investigators assumed standard deviation of VFD at 28 days as SD = 9. Using these assumptions, a total of 142 patients per treatment arm would be needed; after adjustment for drop-out rate, 150 per treatment arm (300 patients per study) will be enrolled.

### Analysis of efficacy

Primary efficacy endpoint (number of VFDs at day 28) will be calculated for both treatment arms separately with corresponding 95% confidence intervals. Comparison between arms will be based on parametric or non-parametric test (following the type of data distribution) and will be adjusted for relevant baseline covariates (stratification parameters).

Secondary and exploratory efficacy endpoints will be analysed following the type of data (chi-square test or Fisher’s exact test for binary data and ANOVA or a non-parametric alternative tests for continuous and ordinal data, if appropriate).

The primary population for analysis of efficacy will be intention-to-treat population (ITT), and the results will be confirmed on the per-protocol population (PP).

#### Subgroup analysis

Pre-planned subgroup analysis will be performed regarding the primary outcome variable in the subgroups defined by the following criteria:

- Age
- Sex
- BMI
- Comorbidities
- P/F ratio
- Length of dexamethasone treatment before enrolment
- ECMO procedure
- Other corticosteroids than study medication administered (from day 11 to day 28)

No adjustments of *p* values due to multiplicity are planned.

### Analysis of safety

The safety will be analysed in all patients who received any dose of study treatment and provided at least one post-dose safety assessment (safety population). All adverse events will be coded and tabulated by system organ class and preferred term for individual events within each system organ class and will be presented in descending frequency. Adverse events will also be tabulated by severity and relationship to the study medication. Serious adverse events will be summarized separately.

### Planned interim analysis

One interim analysis is planned for the safety and efficacy evaluation after the primary outcome is known for the 150th subject (50% of planned sample size). To control the overall level of type I error, the *p* value of 0.01 for interim analysis and 0.04 for the final analysis will be applied. All the details on stopping rules for interim analysis will be described in SIAP.

The interim analysis will be evaluated, and the decision about continuation of the study will be done by DMC.

### Missing data

No imputation techniques for missing data will be applied.

## Data management and quality assurance

### eCRF database

All participants will be assigned an identification code to ensure the pseudonymization of their data. The investigator will maintain a subject identification list for the trial centre (subject identification codes with the corresponding subject names) to enable records to be identified.

Trial data will be collected in eCRFs managed in electronic data capture system REDCap. Access to the database (username, password) will be granted by the study data manager, and the respective study staff will be trained for using it right and safely. The investigator is responsible for the data correctness, completeness, and filling in time. eCRF will be designed to generate queries on missing data or unusual data entries, which will be fed back to the study site investigators in regular intervals.

### Trial documents and medical records

The sponsor, the trial site and the study staff will handle the subject’s personal and trial data according to the effective legislation regarding data protection. Any paper or electronic trial documents or data are confidential and must not be disclosed to the third persons. In the informed consent form, the participants are informed that their medical records can be provided only to the authorized monitors, auditors or inspectors.

Medical records of the subjects will be retained in the trial site for 15 years (from the end of the clinical trial), so will be the relevant trial administrative documents at the sponsor’s side.

### Monitoring and auditing

The trial centre will be monitored according to the monitoring plan. The objectives of the monitoring are to ensure that the trial participant’s safety and rights are respected, that accurate, valid and complete data are collected and that the trial is conducted in accordance with the trial protocol, the principles of GCP and national legislation. The investigator agrees that the monitor will regularly visit the trial centre and will be given appropriate support (e.g. the access to all necessary documents including patient’s medical records). A report on the progress, findings and resolution of any discrepancies will be prepared from each monitoring visit. The investigator undertakes to read the monitoring report and to ensure that any possible discrepancies are corrected. The sponsor, regulatory authority and ethics committees have the right to inspect/audit the trial site. The investigator undertakes to co-operate will be the inspectors/auditors.

#### Deviations and violations

The following issues are considered as violations to the trial protocol:

- Other corticosteroid than dexamethasone administered (from day 1 to day 10)
- Discrepancy in the informed consent procedure
- Assessment of inclusion/exclusion criteria ex post

The following issues are considered as deviations to the trial protocol:

- Different single dose of dexamethasone administered (from day 1 to day 10)
- Administration of dexamethasone shorter than 10 days (this does not apply for discharging the subject from hospital due to clinical improvement)
- Prolongation of dexamethasone treatment (i.e. number of days on treatment in both arms)
- Administration of other corticosteroids than study medication (from day 11 to day 28)

### Steering committee

The steering committee is constituted by all study investigators of the REMED trial. It is responsible for the development of the study protocol, continuous and final result interpretations and manuscript preparation. The steering committee closely collaborates with independent data monitoring committee and the sponsor.

### Data monitoring committee

Members of the data monitoring committee (DMC) are experts in intensive care medicine which are not involved in the REMED trial and an independent statistician. DMC is responsible for performing the interim analysis and for providing recommendations to the steering committee and the sponsor regarding the safety and continuation of the trial based on evidence of possible significant differences between intervention and control group. Members of DMC are listed in a separate document.

All the documents and additional information regarding data management procedures can be found on the official website of the REMED trial (https://czecrin.cz/projekty/kh-remed/) or can be requested on the following mail: ctc.czecrin@med.muni.cz

## Ethical aspects

This trial will be conducted following the applicable legislation and requirements for good clinical practice according to the ICH E6(R2). Compliance with this standard provides public assurance that the rights, safety and well-being of trial participants are protected and that the clinical trial data are credible. All essential trial documents and their potential amendments will be submitted to the relevant ethics committee and regulatory authority for approval.

### Informed consent procedure

The investigator assesses the patient’s ability to decide and extent of potential consciousness impairment based on GCS and other appropriate clinical measures (at discretion of the trial centre).

#### Fully conscious and oriented patients (GCS 15)

Patient with decision-making capacity will go through the standard procedure (informative interview with the investigator, written information for the patients, the possibility to ask questions and adequate time to discuss with family and decide). If the patient wishes to participate, he/she will provide written prospective informed consent.

#### Patients with limited ability to decide (GCS 14 or 13)

Some patients may be limited in their decisional capacity due to their acute health status, or medication. Generally, if a patient understands simplified information and can communicate verbally, the simplified procedure of obtaining informed consent will be applied. The shortened (one-page) information sheet and consent form for signature will be used.

As soon as the patient regains full decisional capacity, he/she will be approached to provide consent with the continuation of his/her participation in the trial. Patients will be informed about the option to withdraw from the trial. Patients who decide to terminate their involvement can permit the sponsor to use the data collected, or they can ask for deleting all data collected. Both options will be presented to them.

If the patient does not regain a decisional capacity, the initial consent will remain valid.

#### Patients lacking the capacity to decide (GCS 12 or less)

It is expected that a significant proportion of screened patients will lack the capacity to provide informed consent due to severely altered consciousness, severe respiratory distress or sedation necessary to facilitate mechanical ventilation. In this situation, the deferred consent policy will be applied. Such a patient will be enrolled after independent physician witnesses (in writing) that the patient cannot give his/her consent and fulfils eligibility criteria.

Patient’s close person (spouse/partner, close relative, caregiver) will be informed about the patient’s enrolment and the nature of the study. If possible and compliant with the epidemiological restrictions by the government, patient’s close person will meet the investigator for an informative interview, to obtain the information leaflet, and to sign a confirmation that he/she was informed about the patient’s participation in the trial.

As soon as the patient regains decisional capacity, he/she will be approached to provide consent with the continuation of his/her participation in the trial. Patients will be informed about the option to withdraw from the trial. Patients who decide to terminate their involvement can permit the sponsor to use the data collected, or they can ask for deleting all data collected. Both options will be presented to them.

If the patient does not regain a decisional capacity, the initial consent by an independent physician will remain valid.

### Supervision of the informed consent procedure

The process of obtaining informed consent from a patient or an independent physician must always be appropriately documented by the investigator through valid forms and the patient’s medical records, as well. The clinical trial monitor will check the process during the monitoring visits. Important deviations in the process will lead to the termination of the patient’s participation in the trial.

### Vulnerable population

Beside patients with diminished decision capacity, other specifically vulnerable participants (children, pregnant women, prisoners, refugees, institutionalized patients, patients with severe mental illnesses, etc.) will not be enrolled in this clinical trial.

## Publication policy

The results of this clinical trial are planned to be published in the medical literature. Any publications must be approved by the sponsor and meet the quality requirements for current clinical research publications (SPIRIT reporting guidelines [18] and CONSORT statement). The collected data will be shared with other ongoing clinical trials on the same topic for individual patient’s data (IPD) meta-analysis or shared upon relevant requests. Also, de-identified participant-level dataset will be made available 6 months after the publication of the results of the study at www.mendeley.com

## Financing and insurance

REMED is an investigator-initiated clinical trial. Funding will be granted from the project research infrastructure Czech Clinical Research Infrastructure Network CZECRIN (LM 2018128) and University Hospital Brno. Trial funders have no role in the study design, collection, analysis and interpretation of the data. Investigators declare no financial or non-financial competing interests regarding the focus of this trial.

Mandatory insurance of the participants is arranged. The coverage for damages or harm emerging from the participation in the clinical trial will be provided according to the applicable legal requirements and from the arranged insurance of the participants.

## Data Availability

The sponsor, the trial site and the study staff will handle the subject’s personal and trial data according to the effective legislation regarding data protection. Collected data will be shared with other ongoing clinical trials on the same topic for individual patient data (IPD) meta-analysis or shared upon relevant requests. A de-identified participant-level dataset will be made available 6 months after the publication of the results of the study at www.mendeley.com

## Footnotes

1 The sponsor expects a high mortality rate due to the natural course of the disease in patients that need ICU care. Relatedness of death to dexamethasone use will be assessed by the trial investigator. Cases considered “not related” will not be separately reported by the sponsor to the RA/EC. The sponsor will be notified of them through eCRF records, and RA/EC will be informed by the sponsor through periodical Annual Reports and final CSR. Cases of death considered “related” will be handled as SAE at the trial site and reported separately and immediately by the sponsor to the RA/EC.
